# Targeted next-generation sequencing in sepsis-associated lymphopenia: a concordance analysis with conventional microbiological testing

**DOI:** 10.3389/fcimb.2026.1938021

**Published:** 2026-09-17

**Authors:** Yifei Xu, Jie Ren, Yue Zheng, Lifeng Huang, Yawen Xie, Na Cui

**Affiliations:** Department of Critical Care Medicine, Beijing Chao-Yang Hospital, Capital Medical University, Beijing, China

**Keywords:** antibiotic stewardship, diagnostic technologies, lymphopenia, sepsis, targeted next-generation sequencing

## Abstract

**Objective:**

This study aimed to evaluate the concordance between targeted next-generation sequencing (tNGS) and conventional microbiological tests (CMT), and to investigate whether tNGS-CMT concordance varies with the degree of immune impairment in patients with sepsis-associated lymphopenia.

**Methods:**

Between October 2024 and March 2026, this study enrolled 93 patients with sepsis-associated lymphopenia who were treated at Peking Union Medical College Hospital and Beijing Chaoyang Hospital. Specimens from clinically suspected infection sites were collected from these patients, and both tNGS and CMT were performed. The detection rate, results, and turnaround time of the two methods were compared, and the association between host immune status, as indicated by lymphocyte subset counts, and tNGS-CMT concordance was examined.

**Results:**

Among the 93 tNGS tests, 80 were positive, while 211 of the 588 CMTs were positive. Comparison of the results revealed complete concordance between tNGS and CMT in 30 of 62 comparable cases (48.4%) and partial concordance in 16 of 62 cases (25.8%). Greater concordance between tNGS and CMT results was significantly associated with severe immune impairment, as indicated by lower CD4+ T-cell counts.

**Conclusion:**

In patients with sepsis-associated lymphopenia, tNGS of specimens from multiple sites demonstrated a higher positivity rate and shorter turnaround time. Furthermore, patients with more severe immune impairment exhibited a higher tNGS-CMT concordance, as indicated by their lower CD4+ T-cell counts.

## 1 Introduction

Sepsis remains a serious problem in intensive care units (ICUs) worldwide, as it is one of the primary causes of in-hospital mortality and complications among critically ill patients (Rudd et al., 2020). Early identification of potential pathogens is crucial for timely antimicrobial decision-making (Kumar et al., 2006), particularly in patients with sepsis-associated lymphopenia. Lymphopenia indicates that the host’s immune function is compromised, which is associated with an elevated risk of secondary infection, persistent organ dysfunction, and adverse outcomes. Infection in these patients is often more complex; they require more rapid and reliable pathogen testing and belong to a high-risk group for infection.

In clinical practice, conventional microbiological tests (CMT) are commonly used to diagnose pathogens. However, these methods have disadvantages, including a long test cycle, limited sensitivity, and susceptibility to interference from factors such as low pathogen loads and atypical pathogens. These limitations may complicate timely etiological assessments and antimicrobial decision-making (Papamentzelopoulou et al., 2025). Next-generation sequencing (NGS) has recently provided a novel approach for pathogen detection. Targeted NGS (tNGS) is one such technology. It integrates targeted amplification with high-throughput sequencing technology and offers high sensitivity, a short detection turnaround time, and low cost. However, tNGS has limited ability to identify pathogens because it relies on a preset genetic detection panel (Hassall et al., 2024; Papamentzelopoulou et al., 2025).

The way tNGS-CMT concordance varies across different degrees of immune impairment in patients with sepsis-associated lymphopenia remains unclear. In this study, we compared pathogen detection using tNGS and CMT at clinically suspected infection sites and investigated the correlation between host immune status and tNGS-CMT concordance. We used lymphocyte subset parameters to characterize host immune status. These findings may help clarify the relationship between host immune status and microbiological test concordance in this population.

## 2 Materials and methods

### 2.1 Research design and research subjects

This study is a dual-center, real-world, observational study. Patients with sepsis (diagnosed according to the Sepsis-3 criteria) who underwent tNGS in the Department of Critical Care Medicine at Peking Union Medical College Hospital and Beijing Chaoyang Hospital from October 2024 to March 2026 were enrolled consecutively. The primary objectives of this study were to characterize pathogen detection by tNGS at clinically suspected infection sites and to explore the relationship between host immune status and tNGS-CMT concordance in patients with sepsis-associated lymphopenia. Inclusion criteria were as follows: (1) Age ≥18 years old; (2) ICU stay > 24 hours; (3) New-onset lymphopenia following sepsis diagnosis, lymphocyte count ≤1.2×10^9^/L (Drewry et al., 2014). Exclusion criteria: (1) Missing clinical data (including microbiology data); (2) Suffering from primary or acquired immunocompromised states due to hematological disorders, immunodeficiency disorders, chemotherapy, and glucocorticoid treatment within the past six months preceding ICU admission; (3) Expected survival less than 24 hours; (4) No written informed consent was obtained. This study was approved by the Medical Ethics Committees of Peking Union Medical College Hospital (No. I-22PJ1104) and Beijing Chaoyang Hospital (No. 2025-869) and was conducted in accordance with the principles of the Declaration of Helsinki.

### 2.2 Data collection

In this study, patient demographic and medical information, and the type and location of infection, were collected. The Acute Physiology and Chronic Health Evaluation II (APACHE II), Sequential Organ Failure Assessment (SOFA), and ICU mortality were also documented. Within 24 h of ICU admission, specimens were collected from clinically suspected infection sites, including peripheral blood, sputum, bronchoalveolar lavage fluid (BALF), cerebrospinal fluid (CSF), and drainage fluid, according to the suspected site of infection and clinical indications. Respiratory infections were further classified as community-acquired pneumonia (CAP), hospital-acquired pneumonia (HAP), or ventilator-associated pneumonia (VAP) according to the American Thoracic Society/Infectious Diseases Society of America (ATS/IDSA) criteria (Kalil et al., 2016; Metlay et al., 2019). CAP was defined as pneumonia that presented in the community or at hospital admission with compatible clinical and radiological findings. HAP was defined as pneumonia that developed ≥48 h after admission and was not associated with mechanical ventilation. VAP was defined as pneumonia that developed >48 h after endotracheal intubation. BALF was collected in intubated patients with suspected pulmonary infection for microbiological testing of the lower respiratory tract. These samples were used for tNGS analysis, conventional rapid tests (Gram-stained smears), and culture. The time of sampling and the time at which valid test results were issued were recorded.

### 2.3 Lymphocyte subsets and serum inflammatory markers

Routine laboratory tests, including complete blood count, C-reactive protein, procalcitonin, and immune markers, were performed upon ICU admission. Flow cytometry was performed using a three-color EPICS-XL flow cytometer (Beckman Coulter, Brea, CA), and the data were processed using System II software (Beckman Coulter). Lymphocyte populations were gated based on forward scatter (FSC) and side scatter (SSC) parameters. This study quantified the following lymphocyte subsets: CD3+ T-cells, CD4+ T-cells (CD3+CD4+), CD8+ T-cells (CD3+CD8+), B-cells (CD19+), and natural killer (NK) cells (CD3-CD16+CD56+).

### 2.4 tNGS and data analysis

tNGS was performed using a pre-specified pathogen panel (**Supplementary Table 3**).

Specimen preparation and nucleic acid extraction: Each sample was first preprocessed. Different sample types have different preprocessing steps.

Bronchoalveolar lavage fluid: The viscous sample was first diluted 5-fold in 1 × phosphate-buffered saline (PBS). A total of 1.7 mL of the diluted sample was centrifuged at 16,000 × g for 5 min. After centrifugation, the supernatant was collected, and the pellet was resuspended separately. Nucleic acid protection solution was added to the pellet, and mechanical disruption was performed. After crushing, the sample was centrifuged at 10,000 × g for 2 min. The supernatant was collected, combined with the previously obtained supernatant, and diluted to 200 μL.

Thoracic and ascitc fluids and drainage fluid: A 5.1 mL sample was evenly distributed in three centrifuge tubes and centrifuged at 16000 × g for 5 min. After centrifugation, the supernatant and resuspended pellets were collected from each tube. All pellets were combined, nucleic acid protection solution was added, and the mixture was mechanically disrupted. After crushing, the sample was centrifuged at 10,000 × g for 2 min. The supernatant was collected, mixed with the previously collected supernatant, and diluted to 200 μL. A nucleic acid extraction kit (YGZZ017; Yuguo Biotechnology (Beijing) Co., Ltd.) was used to extract DNA and RNA from a processed sample (200 μL). The obtained RNA was subsequently reverse transcribed using a first-strand cDNA synthesis kit (YG-048; Yuguo Biotechnology (Beijing) Co., Ltd.).

Target enrichment, library construction, and sequencing: A multiplex pathogen detection kit (YG-032; Yuguo Biotechnology (Beijing) Co., Ltd.) was used for nucleic acid target enrichment. The kit was based on the multiprime primer design platform. The sequencing library was constructed using a universal sequencing reaction kit (YGZZ018; Yuguo Biotechnology (Beijing) Co., Ltd.). Single-end 75-bp sequencing was performed using an Illumina NextSeq 550Dx sequencer (Illumina, USA). Each experimental batch included a no-template control (NTC) that was sterile water instead of the sample.

Bioinformatics analysis process: Fastp (version 0.20.0) was used to filter adapter sequences and low-quality reads from the raw sequencing data using the default parameters. The reads were aligned to the human reference genome (GRCh38.101), and host-derived sequences were removed using Bowtie2. The remaining sequences were compared with a local database using BLAST, which was sourced from NCBI RefSeq and GenBank. The pathogen was identified using these findings and primer information. A pathogen was considered positive only when its organism-specific RPM (tiered by microbial category; **Supplementary Table 3**), primer-pair detection proportion, and sample-to-NTC RPM ratio simultaneously met the pre-specified thresholds. Mixed infection was defined as the detection of two or more pathogens that satisfy the pre-specified positivity criteria within the same specimen (Guan et al., 2025).

### 2.5 Statistical analysis

Statistical analyses were performed using R software (version 4.5.0). Continuous variables are presented as mean ± standard deviation or median (interquartile range), as appropriate. These variables were compared using the independent-samples t-test or the Mann–Whitney U test. Categorical variables are presented as counts (%) and were compared using the chi-squared or Fisher’s exact tests.

Lymphocyte parameters were compared among the three tNGS-CMT concordance groups using the Kruskal–Wallis test, with Bonferroni correction applied for the six lymphocyte parameters. Significant variables were further assessed using Dunn’s *post hoc* test with Bonferroni adjustment. In the subsequent exploratory analyses, following exploratory screening of the six lymphocyte parameters, the CD4+ T-cell count was used as a representative marker of host immune impairment. Receiver Operating Characteristic (ROC) analysis was performed to further characterize the relationship between the degree of immune impairment (indicated by the CD4+ T-cell count) and tNGS-CMT concordance (partial/complete match versus mismatch). The area under the curve (AUC) with 95% confidence interval (CI), sensitivity, specificity, Positive Predictive Value (PPV), and Negative Predictive Value (NPV) were reported. The Youden index was used to establish an exploratory threshold. Internal stability was assessed using 2,000 bootstrap resamples.

A multivariable logistic regression model was used to assess whether the association between the degree of immune impairment (represented by the CD4+ T-cell count) and tNGS-CMT concordance persisted after adjustment for previous antibiotic exposure, age, APACHE II score, and comorbidity burden. The adjusted odds ratio (ORs) with 95% CIs were reported. Treatment outcome analyses were considered exploratory. Agreement between tNGS and CMT was assessed using Cohen’s kappa coefficient for overall and specimen-specific 2×2 categorical comparisons, with percentage agreement additionally reported when marginal distributions were imbalanced. Continuous variables with skewed distributions were summarized as median (IQR) and compared using the Wilcoxon rank-sum test. For ICU length of stay, ICU mortality was considered a competing event and cumulative incidence functions with Gray’s test were additionally performed. A two-sided *P* < 0.05 was considered statistically significant.

## 3 Results

### 3.1 General characteristics of patients and sources of specimens

Based on the inclusion and exclusion criteria (**Figure 1**), 93 patients with sepsis-associated lymphopenia were included in this study cohort. The baseline data of all enrolled patients are presented in **Table 1**. The distribution of patients, baseline characteristics between the two participating centers were comparable (all P>0.05; **Supplementary Table 4**). Specimens for tNGS and CMT were obtained from clinically suspected infection sites, including plasma, sputum, BALF, CSF and drainage fluid (**Figure 2**).

**Figure 1:**
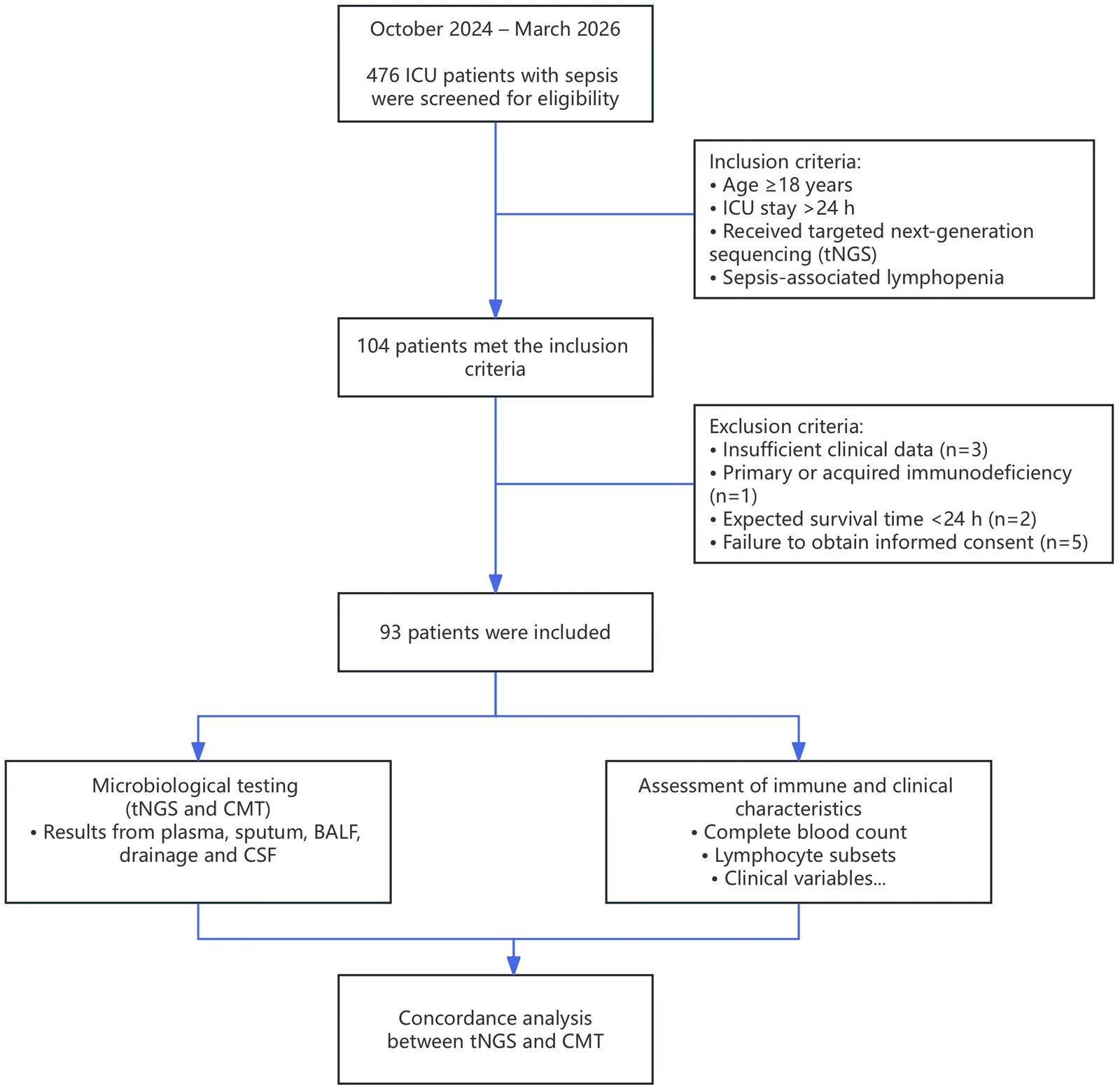
Flowchart.

**Table 1:** The general characteristics of the patients included in this study.

Characteristics	n=93
Age, yr	66.2 ± 13.8
Sex, n (%)	
Male	68 (73.1%)
Total lymphocyte count (cells/mm^3^)	590 (440–930)
T lymphocytes (cells/mm^3^)	319 (215–484)
CD4+ T lymphocytes (cells/mm^3^)	176 (108–274)
CD8+ T lymphocytes (cells/mm^3^)	120 (66–189)
B lymphocytes (cells/mm^3^)	83 (30–144)
NK cells (cells/mm^3^)	45 (20–73)
Comorbidities, n (%)	
Heart failure	24 (25.8%)
Diabetes mellitus	33 (35.5%)
Chronic kidney disease	19 (20.4%)
Coronary artery disease	35 (37.6%)
Rheumatic disease	4 (4.3%)
SOFA	6.7 ± 4.5
APACHE II	15.9 ± 7.4
ICU mortality	15 (16.1%)

**Figure 2:**
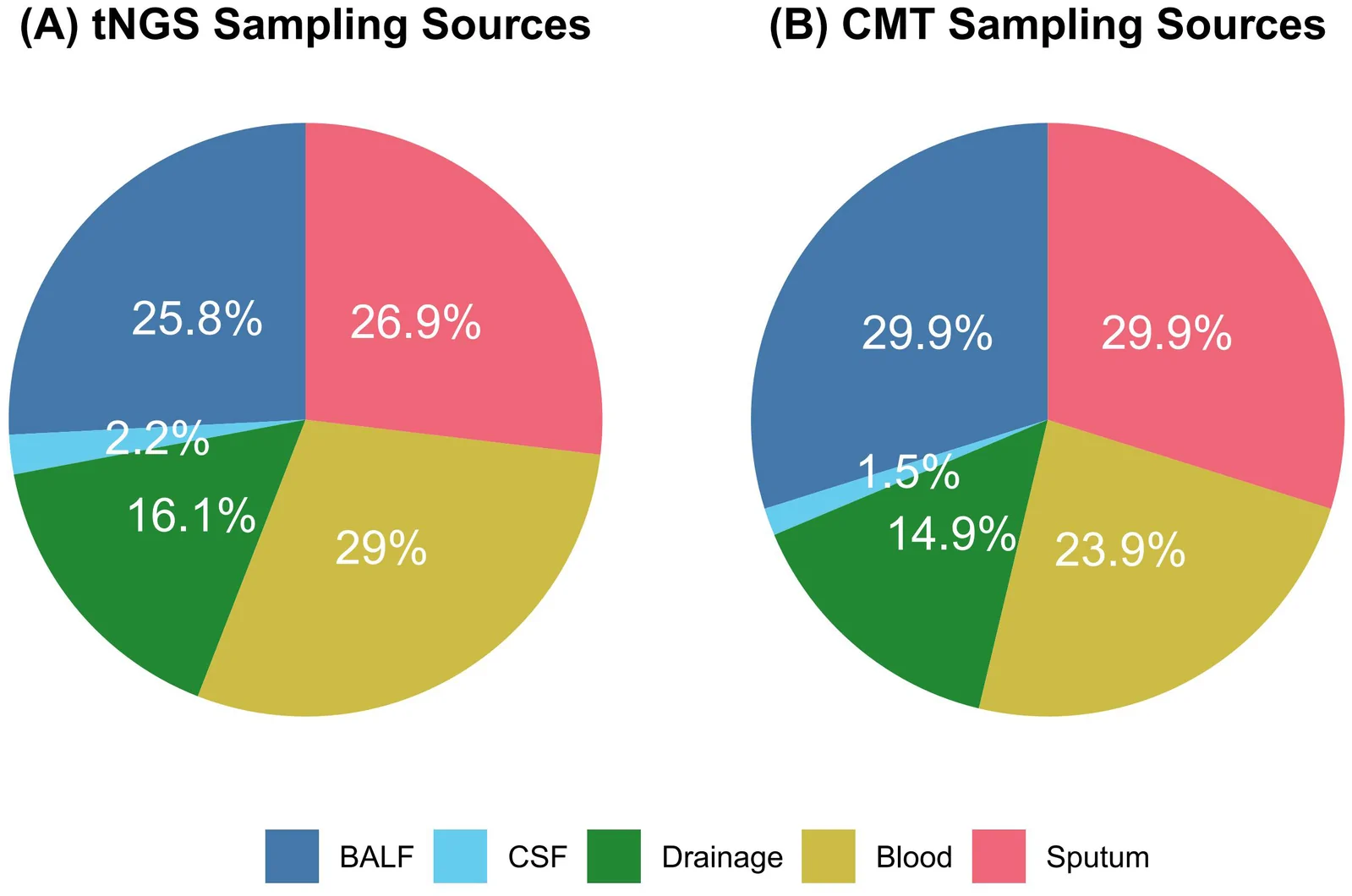
Sampling sources analyzed by tNGS and CMT and distribution of clinically suspected infection sites. **(A)** Distribution of tNGS sampling sources. **(B)** Distribution of CMT sampling sources.

### 3.2 Pathogen detection distribution by tNGS and CMT

Among the 93 tNGS test samples, 80 were positive for pathogens, while 211 of the 588 CMT samples were positive for pathogens. The pathogens identified by tNGS were categorized by organism class (**Figure 3**). Among all specimen types examined, Gram-negative bacteria were identified more frequently than Gram-positive bacteria. Among the Gram-negative bacteria, *Acinetobacter baumannii*, *Klebsiella*, and *Pseudomonas aeruginosa* were the most frequently observed, whereas *Enterococcus* was the most frequently observed Gram-positive bacterium.

**Figure 3:**
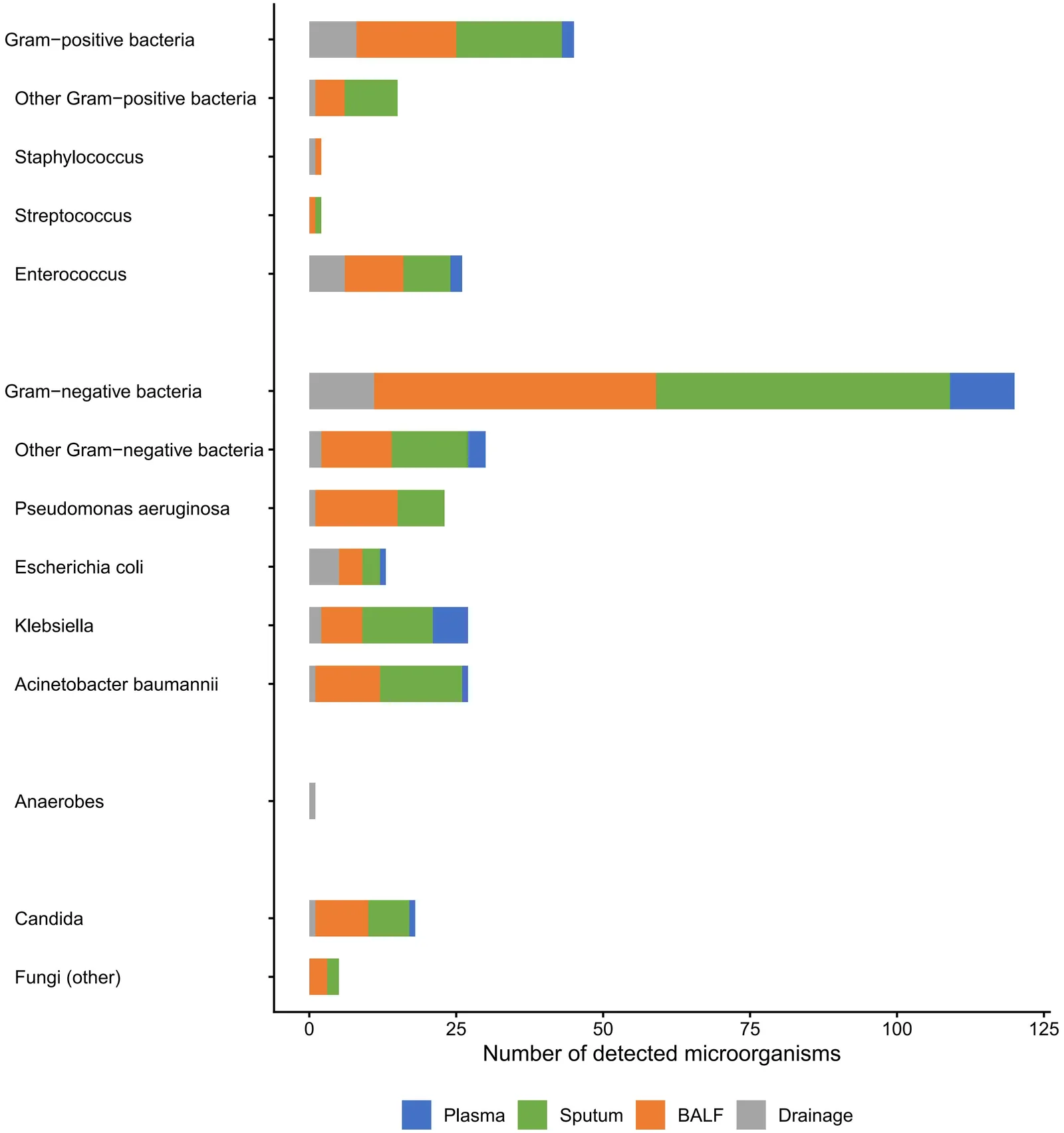
Distribution of tNGS pathogens identified across different sampling sources. BALF, bronchoalveolar lavage fluid. CSF specimens (n = 2) are not shown, as the extremely small sample size precluded a meaningful distributional comparison.

A comparison of the positive detection rates (**Figure 4A**) demonstrated that tNGS exhibited higher positivity rates than CMT in plasma (63.0% versus 61.5%), sputum (100.0% versus 80.0%), BALF (100.0% versus 87.5%), and drainage fluid specimens (93.3% versus 78.6%). For CSF specimens, the positivity rates were 0% for tNGS and 50.0% for CMT. However, only two CSF specimens were available, precluding meaningful comparison. Wilson 95% confidence intervals were calculated for the positivity rate of each method within each specimen type, and paired McNemar’s tests were performed to compare tNGS and CMT positivity in the same patients; no significant differences were observed for any specimen type (all P ≥ 0.07; **Supplementary Table 5**).

**Figure 4:**
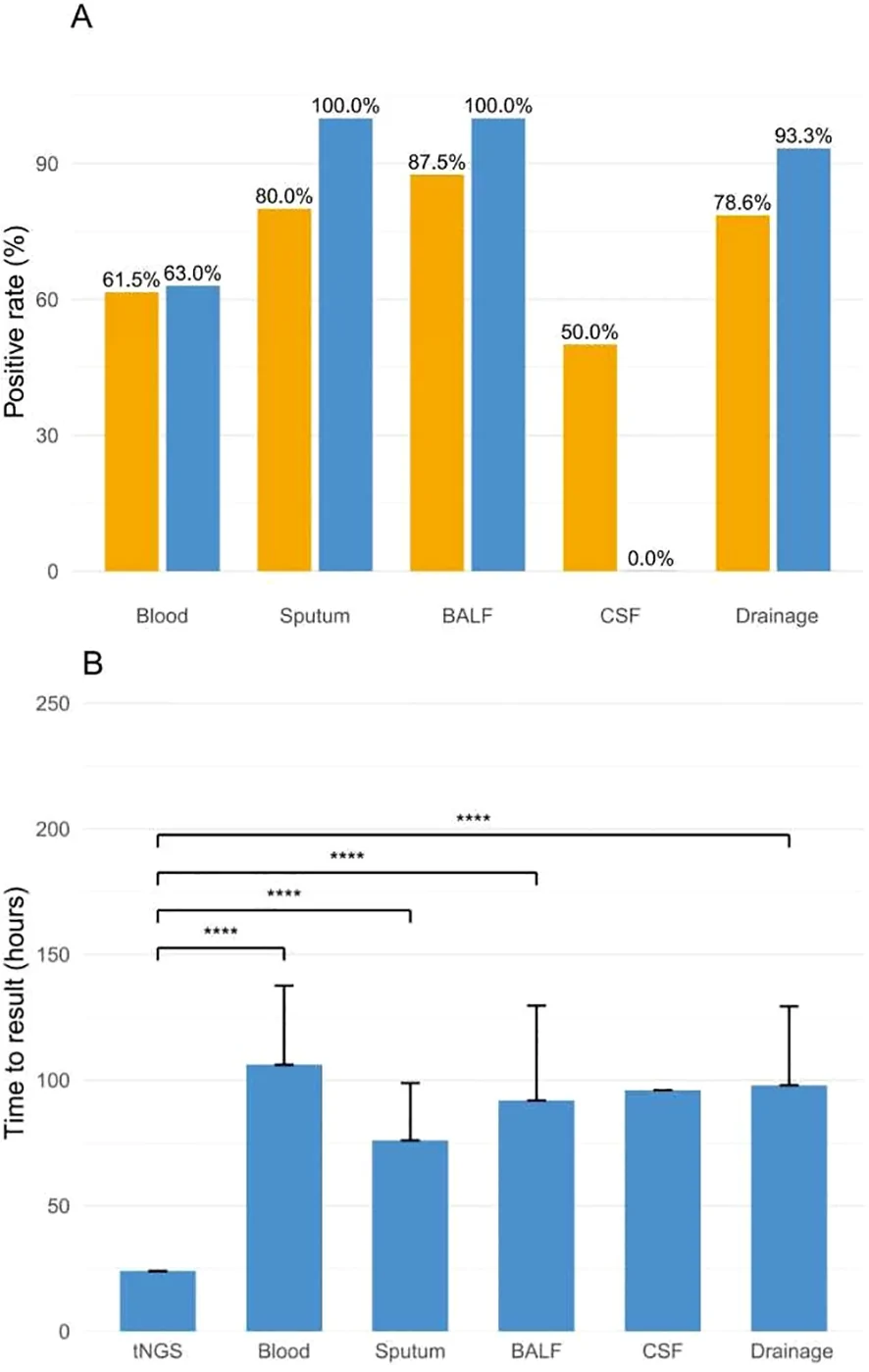
Comparison of positivity rates and reporting times across specimen types. **(A)** Positive rates of CMT (orange) and tNGS (blue) by specimen type, compared using paired McNemar’s test. **(B)** Turnaround time for tNGS versus CMT, presented as mean ± SD and compared using the Wilcoxon rank-sum test with Holm’s correction; ****, Holm-adjusted P < 0.0001. tNGS, targeted next-generation sequencing; CMT, conventional microbiological test; BALF, bronchoalveolar lavage fluid; CSF, cerebrospinal fluid.

Overall agreement between tNGS and CMT was 78.0% (Cohen’s κ = 0.291, P = 0.003; **Supplementary Table 5**). For specimen types with marked marginal imbalance and limited sample sizes (particularly sputum, BALF, and CSF), κ values were interpreted together with percentage agreement because of the known influence of marginal distribution imbalance on kappa statistics.

The turnaround time of tNGS was significantly shorter than that of conventional culture (**Figure 4B**). The mean reporting time for tNGS was 24.0 h, compared with 106.0 ± 31.5 h for blood cultures, 76.0 ± 22.8 h for sputum cultures, 91.9 ± 37.8 h for BALF cultures, and 97.9 ± 31.4 h for drainage fluid cultures. All comparisons with tNGS were statistically significant (all *P* < 0.0001). Only one CSF culture result was available (96 h); therefore, this specimen type was excluded from the statistical comparison.

### 3.3 Comparison between tNGS and CMT

To assess the consistency between tNGS and CMT results, we compared the two tests at the infection site in 93 patients. Of these, 20 samples (21.5%) were excluded because no valid conventional testing method was available as a reference, leaving 73 samples with comparable data (**Figure 5A**). Among these 73 samples, 11 (15.1%) tested negative by both methods, indicating negative concordance. Of the remaining 62 samples, 16 (25.8%) showed completely discordant pathogen detection results with no overlapping pathogens; another 16 samples (25.8%) exhibited partial matches; and 30 samples (48.4%) demonstrated complete pathogen-level concordance (**Figure 5B**).

**Figure 5:**
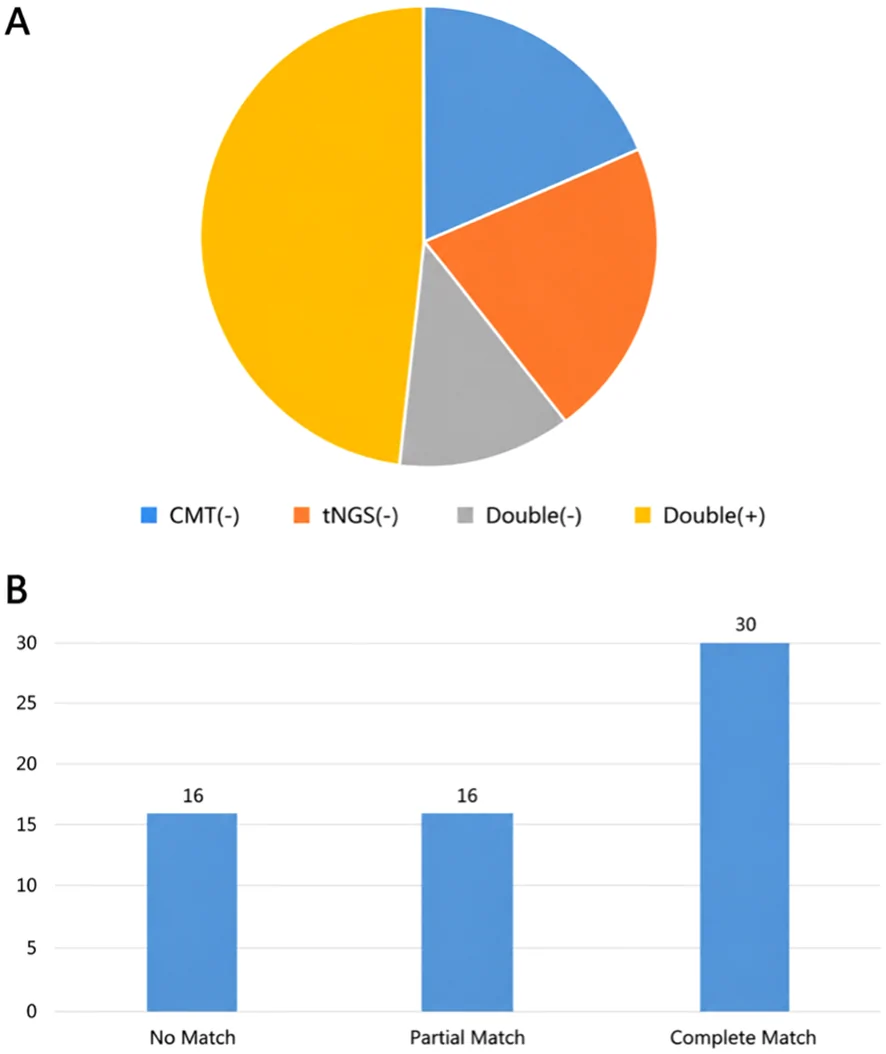
Analysis of number of samples with matching tNGS and CMT results. **(A)** Proportions of tNGS and CMT results for clinically suspected infection sites. **(B)** Concordance of tNGS results for cases with both on-site tNGS/CMT results.

Among the samples included in the positive concordance analysis, the overall positive concordance rate was 74.2%. When stratified by specimen type, the overall concordance rate was highest for BALF specimens (90.0%), followed by sputum (82.4%), plasma (77.3%), drainage fluids (58.3%), and CSF (50.0%). The positive concordance rate followed a consistent pattern, with the highest rate observed in BALF (90.0%), followed by sputum (82.4%), plasma, and drainage fluids (58.3%). However, no positive concordance was observed in CSF specimens (**Table 2**).

**Table 2:** Concordance between targeted next-generation sequencing (tNGS) and conventional microbiological tests (CMT) stratified by specimen type.

Specimen type	Samples (n)	Comparable samples (n)	Discordance n (%)	Overall concordance rate^a^, %	Positive concordance rate^b^, %
Plasma	27	22	5 (22.7)	77.3	58.3
Sputum	25	17	3(17.6)	82.4	82.4
BALF	24	20	2(10.0)	90.0	90.0
Drainage fluids	15	12	5 (41.7)	58.3	58.3
Total	93	73	16 (21.9)	78.1	74.2

BALF, bronchoalveolar lavage fluid; CSF specimens (n =2) are not presented separately in this table because of the very small sample size.

a Overall concordance rate = (full + partial + negative concordance)/comparable samples × 100.

b Positive concordance rate = (full + partial concordance)/(full + partial concordance + discordance) × 100, i.e., concordance restricted to samples in which conventional testing yielded a positive result.

### 3.4 Association between host immune status and tNGS-CMT concordance

To explore the correlation between host immune status and tNGS-CMT concordance, lymphocyte parameters were compared across three concordance groups: mismatch, partial match, and complete match (**Figure 6**).

**Figure 6:**
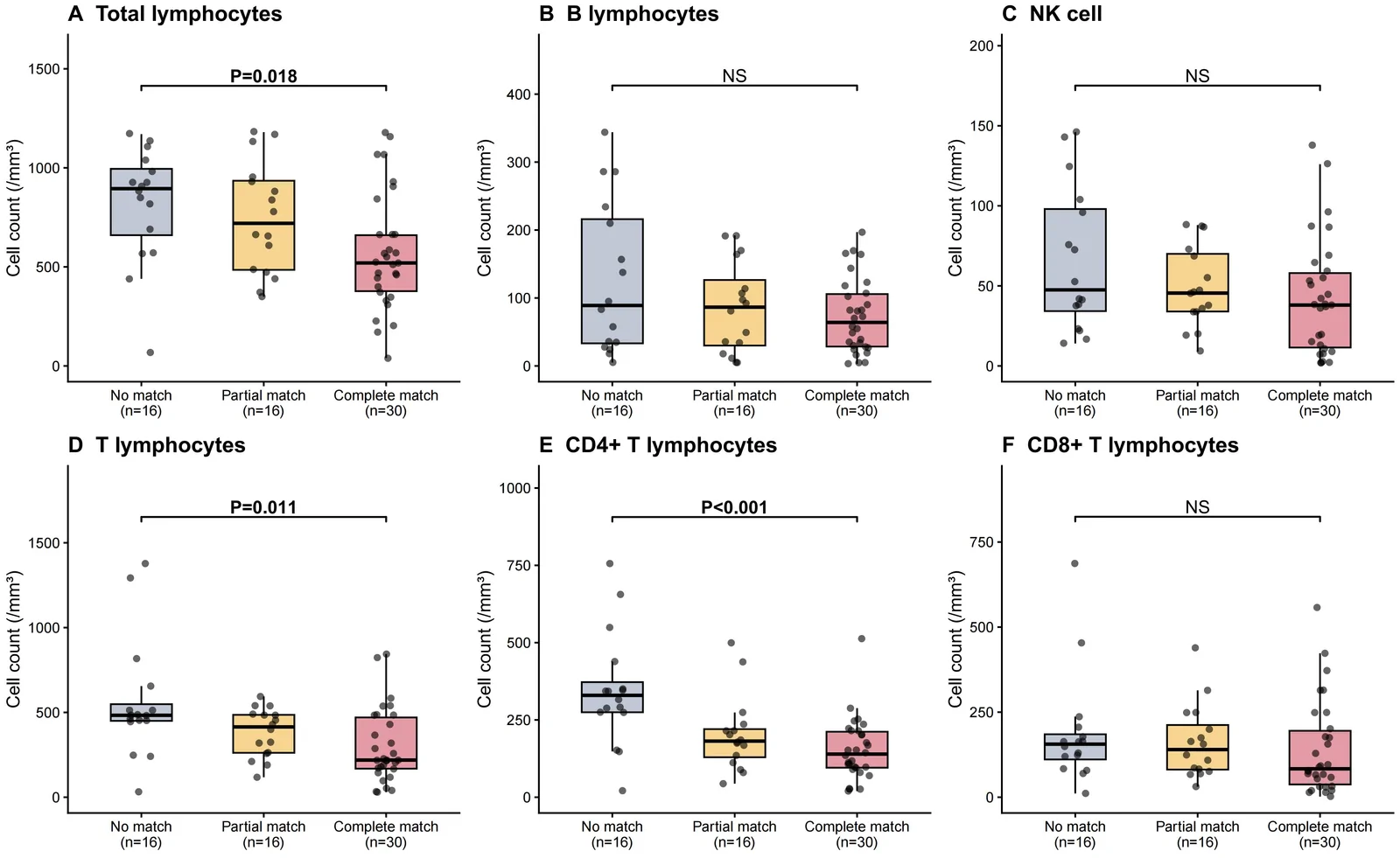
Comparison of peripheral lymphocyte subset counts grouped by the accordance between tNGS and CMT results for clinically suspected infection sites. **(A)** The counts of total lymphocytes, **(B)** B lymphocytes, **(C)** NK cell, **(D)** T lymphocytes, **(E)** CD4+ T lymphocytes and **(F)** CD8+ T lymphocytes in patient groups with no match, partial match, and complete match. NS, nonsignificant.

Kruskal–Wallis analysis revealed nominal differences in total lymphocyte and total T-cell counts among the three groups (total lymphocytes: median 895, 720, and 520 cells/mm^3^, *P* = 0.018; T-cells: median 483, 415, and 219 cells/mm^3^, *P* = 0.011). However, these differences were not statistically significant after Bonferroni correction for the six lymphocyte parameters (adjusted *P* = 0.109 and 0.065, respectively).

Among the six lymphocyte parameters evaluated as candidate markers of host immune status, only the CD4+ T-cell count remained significantly associated with tNGS-CMT concordance after correction for multiple comparisons. The median CD4+ T-cell counts were 330, 182, and 140 cells/mm^3^ in the mismatch, partial match, and complete match groups, respectively (*P* < 0.001; Bonferroni-adjusted *P* = 0.0015). *Post hoc* Dunn’s test with Bonferroni correction revealed that CD4+ T-cell counts in the mismatch group were significantly higher than those in the partial-match (adjusted *P* = 0.038) and complete-match (adjusted *P* < 0.001, **Supplementary Table 1**) groups, whereas no significant difference was observed between the partial-match and complete-match groups (adjusted *P* = 0.669). No significant differences were observed in the number of CD8+ T-cells, B-cells, or NK cells (**Figures 6B, C, F**).

Using CD4+ T-cell count as a representative marker of host immune impairment, exploratory ROC analysis demonstrated an AUC of 0.828 (95% CI: 0.685–0.971) for distinguishing concordant (partial/complete match) from mismatched cases (**Figure 7**). The Youden index identified an exploratory threshold of 253 cells/mm^3^. At this exploratory threshold, the sensitivity, specificity, positive predictive value, and negative predictive value were 89.1%, 81.3%, 93.2%, and 72.2%, respectively. Internal validation using 2,000 bootstrap resamples demonstrated minimal optimism (bootstrap mean AUC 0.830; optimism-corrected AUC 0.826; bootstrap 95% CI: 0.676–0.952), supporting the stability of this exploratory association.

**Figure 7:**
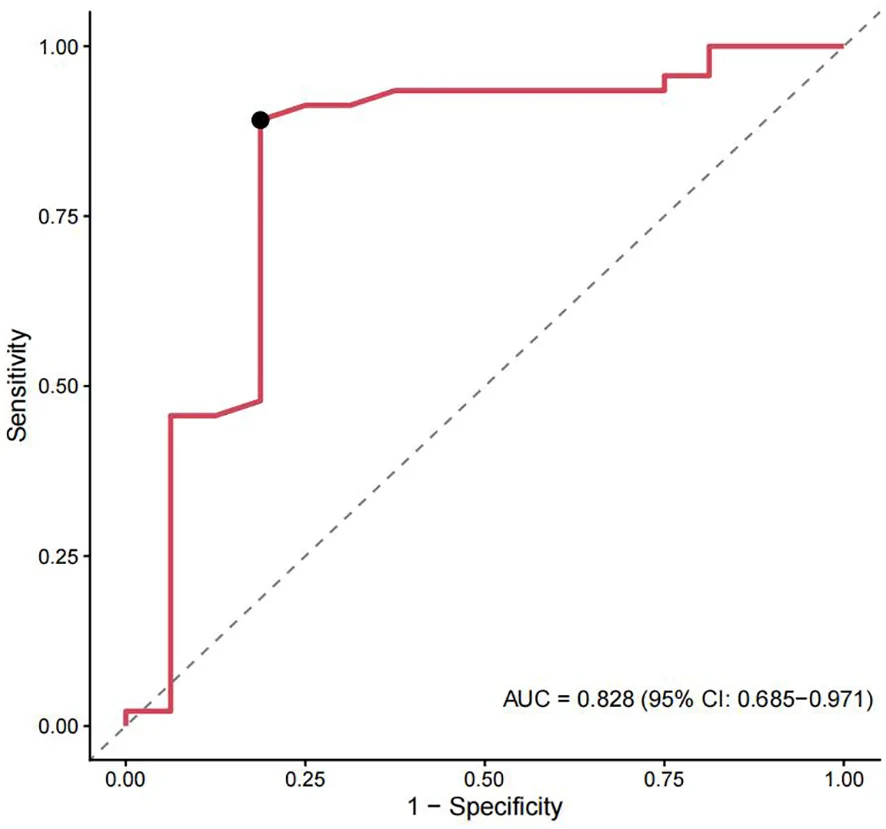
Exploratory receiver operating characteristic (ROC) curve for CD4+ T-cell count in distinguishing concordant (partial/complete match) from mismatched tNGS-CMT results. AUC = 0.828 (95% CI: 0.685–0.971). The Youden index identified an exploratory threshold of 253 cells/mm³ (sensitivity 89.1%, specificity 81.3%).

After adjusting for previous antibiotic exposure, age, APACHE II score, and comorbidity burden, a more significant state of immune impairment (represented by lower CD4+ T-cell counts) in patients with sepsis-associated lymphopenia remained associated with a higher probability of tNGS-CMT concordance (*P* = 0.003). Each 50-cells/mm^3^ decrease in the CD4+ T-cell count corresponded to an adjusted OR of 1.51 for concordance (95% CI: 1.19–2.05; **Supplementary Table 2**).

### 3.5 Association between tNGS-concordant therapy and clinical outcomes

Clinical outcomes were compared in an exploratory treatment outcome analysis based on the concordance of the initial antimicrobial regimen with tNGS results and pathogen class. In the overall cohort, no significant differences in ICU mortality, ICU length of stay, or vasoactive agent duration were observed between the tNGS-concordant and discordant treatment groups for either Gram-positive or Gram-negative bacteria (all *P* > 0.05).

Exploratory analyses were performed among patients with a more significant state of immune impairment, operationally represented by CD4^+^ T-cell count <253 cells/mm³. ICU length of stay and vasopressor duration were compared using the Wilcoxon rank-sum test. No significant differences were observed in Gram-positive infections. In Gram-negative infections, discordant cases had longer ICU stays and vasopressor use (*P* = 0.007 and 0.033, respectively); however, the association with ICU stay was attenuated after competing-risk adjustment for ICU mortality (Gray’s test, *P* = 0.197). ICU mortality did not differ between concordant and discordant groups (**Table 3**).

**Table 3:** Comparison of clinical outcomes stratified by tNGS-detected pathogen type and CD4^+^ T cell count.

Variable	Gram-positive bacteria			Gram-negative bacteria		
	Yes	No	P value	Yes	No	P value
All 80 patients (tNGS-positive)						
N	36	44		56	24	
ICU mortality, %	13.9%	22.7%	0.394	17.9%	20.8%	0.762
ICU length of stay, days	7.0 (4.0)	7.0 (5.2)	0.425	7.0 (4.0)	8.0 (10.0)	0.064
ICU length of stay, competing-risk P			0.432			0.363
Vasoactive agent use, days	2.0 (4.0)	2.0 (4.5)	0.218	2.0 (2.5)	2.0 (11.0)	0.152
58 patients with CD4^+^ T cell count <253 cells/mm³						
N	28	30		38	20	
ICU mortality, %	10.7%	23.3%	0.301	15.8%	20.0%	0.724
ICU length of stay, days	7.0 (3.2)	8.5 (8.2)	0.079	7.0 (5.0)	9.0 (9.0)	0.007
ICU length of stay, competing-risk P			0.161			0.197
Vasoactive agent use, days	1.5 (2.2)	2.0 (10.8)	0.083	1.5 (2.8)	2.5 (11.5)	0.033

tNGS, targeted next-generation sequencing; ICU, intensive care unit; IQR, interquartile range. Notes: Treatment-outcome analyses included 80 tNGS-positive patients, including 58 patients with CD4^+^ T-cell counts <253 cells/mm³. “Yes” indicates that the initial antimicrobial regimen was concordant with subsequent tNGS findings, whereas “No” indicates discordance. The CD4^+^ T-cell cutoff of 253 cells/mm³ was derived from exploratory ROC analysis. Continuous variables with skewed distributions are presented as median (IQR) and compared using the Wilcoxon rank-sum test. A competing-risk analysis using Gray’s test was additionally performed, with in-ICU death treated as a competing event.

## 4 Discussion

The early and accurate identification of pathogens is a fundamental requirement for the clinical management of sepsis. This is particularly important for patients with sepsis-associated lymphopenia who are at a higher risk of secondary and opportunistic infections (Geng et al., 2021). CMT has the disadvantages of a long turnaround time and limited sensitivity, which can delay the etiological diagnosis. This frequently results in the reliance on empirical broad-spectrum anti-infective therapy, which increases the risk of antimicrobial resistance and compromises the delivery of precision treatment (Li et al., 2022; Wang et al., 2024; Liang et al., 2025). Therefore, developing more rapid and all-encompassing pathogen detection technology and clarifying its diagnostic performance across different host immune backgrounds are of great value for improving the prognosis of septic patients.

Recently, the diagnosis of infectious diseases has been revolutionized by the rapid development of high-throughput sequencing technologies, including metagenomic sequencing (mNGS) and tNGS (Brockhurst et al., 2011; Drewry et al., 2014; Rong et al., 2026). Although mNGS offers the advantage of unbiased pathogen identification, its clinical application remains limited by a high host background, contamination risk, complex analytical workflows, and relatively high costs (Chiu and Miller, 2019; Chien et al., 2022; Chen et al., 2024). Targeted enrichment in tNGS may increase analytical detection efficiency and reduce background interference. It has a relatively short turnaround time and may be beneficial for detecting pathogens that are difficult to culture or in settings with multiple pathogen detection (Ballester et al., 2016; Kambli et al., 2024; Yin et al., 2024). However, tNGS has limitations. The preset panel limits the detection range, and important pathogens outside the panel may be overlooked. Consequently, its clinical performance requires further verification. Currently, the academic community has not systematically evaluated the diagnostic importance of tNGS in highly heterogeneous populations, such as those with sepsis, nor has the effect of host immune status on its detection performance been clarified (Yadav et al., 2023).

This study compared the detection results of tNGS and CMT at numerous clinical sites for suspected infection to evaluate the diagnostic performance of this technology in determining the etiology of sepsis-associated lymphopenia. Compared with CMT, tNGS exhibited higher positive detection rates in most specimen types and a significantly shorter turnaround time. The mean reporting time for tNGS was 24 h, compared with 106.0 ± 31.5 h for blood culture, 76.0 ± 22.8 h for sputum culture, 91.9 ± 37.8 h for BALF culture, and 97.9 ± 31.4 h for drainage fluid culture (all *P* < 0.0001). Because only one CSF culture result was available, it was excluded from the statistical comparison. Despite the higher detection yield of tNGS, only 30 of the 62 cases included in the pathogen-level concordance analysis (48.4%) exhibited complete pathogen-level concordance with CMT, indicating that the two methods frequently produced non-identical pathogen profiles. For common Gram-negative bacteria in the ICU, including *Pseudomonas aeruginosa*, *Acinetobacter baumannii, Klebsiella pneumoniae*, and *Escherichia coli*, the consistency between the two assays was relatively high. Most cases with inconsistent results were due to tNGS detecting additional mixed infectious pathogens, opportunistic pathogens, and anaerobic bacteria. The overall concordance rate between tNGS and CMT also varied by specimen source, ranging from 90.0% for BALF and 82.4% for sputum to 77.3% for plasma and 58.3% for drainage fluids. The lower consistency in plasma samples likely results from low pathogen cell-free DNA concentration and possible PCR inhibitors, both of which may reduce the analytical sensitivity of amplification-based detection (Wei et al., 2025). The bacterial load in respiratory specimens is high, and the pathogen spectrum is relatively limited. This attribute is consistent with the conclusion that respiratory specimen consistency in previous studies was generally high (Xu et al., 2025). The consistency of the abdominal specimens was moderate to high, consistent with the reported trend in previous tNGS studies. tNGS can detect mixed infection cases that are completely unrecognizable using traditional culture (Ma et al., 2026). The culture of anaerobic bacteria and some atypical pathogens requires rigorous growth conditions. Traditional culture methods can easily miss detection or require an excessively long detection cycle. tNGS is a nucleic acid detection technology that does not rely on strain survival or require specific culture conditions (Brockhurst et al., 2011; Buhl et al., 2024). It can simultaneously detect multiple pathogens and provide confidence information for the results that can address some limitations of traditional culture. When dominant bacteria are present in mixed-infection samples, traditional culture can easily mask other pathogens. Therefore, tNGS may provide complementary microbiological information in respiratory and abdominal specimens, particularly when multiple microorganisms are identified (Chen et al., 2024).

Previous research on the use of NGS technology for sepsis pathogen diagnosis has primarily focused on the detection efficiency and analytical performance of the sequencing platform. The potential impact of host-pathogen interactions on detection results has rarely been investigated in depth (Fida et al., 2021). This study explored the concordance of tNGS-CMT across different degrees of immune impairment in patients with sepsis-associated lymphopenia. In this study, the CD4+ T-cell count was used only as a representative marker indicating host immune function rather than as an independent diagnostic or predictive biomarker. A more significant state of immune impairment (indicated by lower CD4+ T-cell counts) was associated with a higher tNGS-CMT concordance. These findings suggest that host immune status may influence the interpretation of tNGS-CMT concordance in patients with sepsis-associated lymphopenia, although the underlying mechanisms require further investigation.

In exploratory subgroup analyses, among patients with a more profound state of immune impairment as represented by CD4+ T-cell counts <253 cells/mm^3^, concordance of the initial antimicrobial regimen with tNGS results was associated with shorter vasoactive-agent duration and ICU stay in unadjusted analyses. However, after accounting for ICU mortality as a competing event, the association with ICU stay was attenuated and no longer statistically significant. Therefore, these findings should be interpreted as hypothesis-generating observations rather than evidence of a causal benefit from tNGS-guided antimicrobial management. Further prospective studies are required to determine whether incorporating tNGS information into clinical decision-making can improve patient outcomes.

This study has some limitations. This was a multicenter observational cohort study with a relatively limited sample size, and the findings require further validation in larger and more diverse populations. Because enrollment was restricted to patients who underwent tNGS testing, detailed clinical information was not systematically available for eligible patients who did not receive tNGS, preventing a formal assessment of potential selection bias. In the absence of an independent microbiological reference standard, this study evaluated the concordance between tNGS and corresponding CMT results as the primary analytical framework; therefore, some discordant results could not be definitively adjudicated. Although multivariable analyses were performed, residual confounding related to antimicrobial exposure, disease severity, and clinical decision-making cannot be completely excluded. Furthermore, tNGS findings may be influenced by true polymicrobial infections, colonization, or contamination, and standardized criteria for distinguishing clinically relevant pathogens from non-pathogenic detections remain lacking. In addition, the potential temporal discrepancy between immune status assessment and microbiological specimen collection may affect the interpretation of the observed associations. Finally, whether these findings can be generalized to other populations with immune dysfunction requires further investigation.

## 5 Conclusion

Compared with CMT, tNGS demonstrated higher positive detection rates for most specimen types and a shorter turnaround time in patients with sepsis-associated lymphopenia. Greater tNGS-CMT concordance was observed in patients with more severe immune impairment, as reflected by lower CD4+ T-cell counts. These findings suggest that host immune status may be relevant when interpreting tNGS results, and that tNGS may provide complementary microbiological information when considered alongside CMT and the overall clinical context.

## Data Availability

The data presented in this study are deposited in the CNGB Sequence Archive (CNSA) repository, accession number CNP0009846.
