# Supplementary material for: Targeted next-generation sequencing in sepsis-associated lymphopenia: a concordance analysis with conventional microbiological testing
Source: Front Cell Infect Microbiol. 2026 Sep 17;16:1938021. doi: 10.3389/fcimb.2026.1938021 (PMC13626988; doi:10.3389/fcimb.2026.1938021)
Supplement: Supplementary file 1 [file Table1.docx]

**Supplementary materials**

**Table S1 Pairwise comparisons of CD4+ T lymphocytes**

| Comparison | Z value | P (uncorrected) | P (Bonferroni-corrected) |
| --- | --- | --- | --- |
| Mismatch vs Partial match | 2.49 | 0.013 | 0.038 |
| Mismatch vs Complete match | 4.07 | <0.001 | <0.001 |
| Partial match vs Complete match | 1.22 | 0.223 | 0.669 |

Data were analyzed by Dunn's test with Bonferroni correction.

**Table S2 Multivariable Logistic Regression: Predictors of tNGS–CMT Concordance**

| Variable | OR | 95% CI | P value |
| --- | --- | --- | --- |
| CD4 (per 50-cell/mm³ decrease) | 1.509 | 1.188-2.051 | 0.003 |
| Prior antibiotic use | 0.140 | 0.004-1.565 | 0.177 |
| Age | 1.058 | 1.004-1.127 | 0.051 |
| APACHE II score | 1.040 | 0.941-1.163 | 0.453 |
| Comorbidity count | 0.877 | 0.413-1.919 | 0.733 |

Note: OR, odds ratio; CI, confidence interval.

**Table S3 Complete Target List of the tNGS Panel**

Section A. Complete list of pathogen targets by category (n = 383)

| **No.** | **Category** | **Scientific / English Name** |
| --- | --- | --- |
| 1 | Gram-positive bacteria | Corynebacterium striatum |
| 2 | Gram-positive bacteria | Corynebacterium diphtheriae |
| 3 | Gram-positive bacteria | Corynebacterium urealyticum |
| 4 | Gram-positive bacteria | Enterococcus faecalis |
| 5 | Gram-positive bacteria | Enterococcus faecium |
| 6 | Gram-positive bacteria | Enterococcus avium |
| 7 | Gram-positive bacteria | Enterococcus gallinarum |
| 8 | Gram-positive bacteria | Enterococcus casseliflavus |
| 9 | Gram-positive bacteria | Gemella morbillorum |
| 10 | Gram-positive bacteria | Streptococcus equi |
| 11 | Gram-positive bacteria | Streptococcus sp. |
| 12 | Gram-positive bacteria | Rhodococcus equi |
| 13 | Gram-positive bacteria | Staphylococcus aureus |
| 14 | Gram-positive bacteria | Staphylococcus capitis |
| 15 | Gram-positive bacteria | Staphylococcus epidermidis |
| 16 | Gram-positive bacteria | Staphylococcus haemolyticus |
| 17 | Gram-positive bacteria | Staphylococcus hominis |
| 18 | Gram-positive bacteria | Staphylococcus lugdunensis |
| 19 | Gram-positive bacteria | Staphylococcus equorum |
| 20 | Gram-positive bacteria | Staphylococcus simulans |
| 21 | Gram-positive bacteria | Staphylococcus xylosus |
| 22 | Gram-positive bacteria | Staphylococcus caprae |
| 23 | Gram-positive bacteria | Staphylococcus schleiferi |
| 24 | Gram-positive bacteria | Mammaliicoccus sciuri |
| 25 | Gram-positive bacteria | Staphylococcus arlettae |
| 26 | Gram-positive bacteria | Staphylococcus auricularis |
| 27 | Gram-positive bacteria | Staphylococcus saprophyticus |
| 28 | Gram-positive bacteria | Mammaliicoccus lentus |
| 29 | Gram-positive bacteria | Staphylococcus pseudintermedius |
| 30 | Gram-positive bacteria | Staphylococcus intermedius |
| 31 | Gram-positive bacteria | Staphylococcus argenteus |
| 32 | Gram-positive bacteria | Staphylococcus warneri |
| 33 | Gram-positive bacteria | Staphylococcus spp. |
| 34 | Gram-positive bacteria | Parvimonas micra |
| 35 | Gram-positive bacteria | Rothia dentocariosa |
| 36 | Gram-positive bacteria | Rothia mucilaginosa |
| 37 | Gram-positive bacteria | Streptococcus pneumoniae |
| 38 | Gram-positive bacteria | Streptococcus canis |
| 39 | Gram-positive bacteria | Streptococcus pyogenes |
| 40 | Gram-positive bacteria | Streptococcus suis |
| 41 | Gram-positive bacteria | Streptococcus infantis |
| 42 | Gram-positive bacteria | Streptococcus mutans |
| 43 | Gram-positive bacteria | Streptococcus gordonii |
| 44 | Gram-positive bacteria | Streptococcus sanguinis |
| 45 | Gram-positive bacteria | Streptococcus anginosus |
| 46 | Gram-positive bacteria | Streptococcus constellatus |
| 47 | Gram-positive bacteria | Streptococcus gallolyticus |
| 48 | Gram-positive bacteria | Streptococcus intermedius |
| 49 | Gram-positive bacteria | Streptococcus mitis |
| 50 | Gram-positive bacteria | Streptococcus oralis |
| 51 | Gram-positive bacteria | Streptococcus parasanguinis |
| 52 | Gram-positive bacteria | Streptococcus pseudopneumoniae |
| 53 | Gram-positive bacteria | Streptococcus salivarius |
| 54 | Gram-positive bacteria | Streptococcus vestibularis |
| 55 | Gram-positive bacteria | Streptococcus agalactiae |
| 56 | Gram-positive bacteria | Streptococcus dysgalactiae |
| 57 | Gram-positive bacteria | Actinomyces spp. |
| 58 | Gram-positive bacteria | Actinomyces graevenitzii |
| 59 | Gram-positive bacteria | Actinomyces israelii |
| 60 | Gram-positive bacteria | Actinomyces oris |
| 61 | Gram-positive bacteria | Bacillus cereus group |
| 62 | Gram-positive bacteria | Clostridioides difficile |
| 63 | Gram-positive bacteria | Clostridium perfringens |
| 64 | Gram-positive bacteria | Abiotrophia defectiva |
| 65 | Gram-positive bacteria | Listeria monocytogenes |
| 66 | Gram-positive bacteria | Gordonia bronchialis |
| 67 | Gram-positive bacteria | Abiotrophia defectiva |
| 68 | Gram-positive bacteria | Nocardia asteroides |
| 69 | Gram-positive bacteria | Nocardia farcinica |
| 70 | Gram-positive bacteria | Nocardia abscessus |
| 71 | Gram-positive bacteria | Nocardia veterana |
| 72 | Gram-positive bacteria | Nocardia otitidiscaviarum |
| 73 | Gram-positive bacteria | Nocardia brasiliensis |
| 74 | Gram-positive bacteria | Nocardia asiatica |
| 75 | Gram-positive bacteria | Nocardia brevicatena |
| 76 | Gram-positive bacteria | Nocardia veterana |
| 77 | Gram-positive bacteria | Nocardia beijingensis |
| 78 | Gram-positive bacteria | Nocardia pseudobrasiliensis |
| 79 | Gram-positive bacteria | Nocardia terpenica |
| 80 | Gram-positive bacteria | Nocardia transvalensis |
| 81 | Gram-positive bacteria | Nocardia spp. |
| 82 | Gram-positive bacteria | Clostridium botulinum |
| 83 | Gram-positive bacteria | Clostridium tetani |
| 84 | Gram-positive bacteria | Granulicatella adiacens |
| 85 | Gram-positive bacteria | Tropheryma whipplei |
| 86 | Gram-positive bacteria | Corynebacterium diphtheriae complex |
| 87 | Gram-positive bacteria | Streptococcus mitis group |
| 88 | Gram-negative bacteria | Klebsiella pneumoniae complex |
| 89 | Gram-negative bacteria | Klebsiella pneumoniae |
| 90 | Gram-negative bacteria | Klebsiella aerogenes |
| 91 | Gram-negative bacteria | Klebsiella oxytoca |
| 92 | Gram-negative bacteria | Klebsiella variicola |
| 93 | Gram-negative bacteria | Burkholderia mallei |
| 94 | Gram-negative bacteria | Burkholderia cenocepacia |
| 95 | Gram-negative bacteria | Burkholderia pseudomallei |
| 96 | Gram-negative bacteria | Brucella spp. |
| 97 | Gram-negative bacteria | Brucella melitensis |
| 98 | Gram-negative bacteria | Escherichia coli |
| 99 | Gram-negative bacteria | Escherichia vulneris |
| 100 | Gram-negative bacteria | Haemophilus influenzae |
| 101 | Gram-negative bacteria | Haemophilus parainfluenzae |
| 102 | Gram-negative bacteria | Bacteroides fragilis |
| 103 | Gram-negative bacteria | Pseudomonas sp. (heparin-degrading) |
| 104 | Gram-negative bacteria | Pseudomonas aeruginosa |
| 105 | Gram-negative bacteria | Pseudomonas alcaligenes |
| 106 | Gram-negative bacteria | Pseudomonas putida |
| 107 | Gram-negative bacteria | Aeromonas hydrophila |
| 108 | Gram-negative bacteria | Pseudomonas oryzihabitans |
| 109 | Gram-negative bacteria | Stenotrophomonas maltophilia |
| 110 | Gram-negative bacteria | Comamonas kerstersii |
| 111 | Gram-negative bacteria | Aeromonas caviae |
| 112 | Gram-negative bacteria | Aeromonas sobria |
| 113 | Gram-negative bacteria | Burkholderia cepacia |
| 114 | Gram-negative bacteria | Enterobacter cloacae complex |
| 115 | Gram-negative bacteria | Enterobacter cloacae |
| 116 | Gram-negative bacteria | Enterobacter hormaechei |
| 117 | Gram-negative bacteria | Enterobacter ludwigii |
| 118 | Gram-negative bacteria | Enterobacter asburiae |
| 119 | Gram-negative bacteria | Enterobacter cancerogenus |
| 120 | Gram-negative bacteria | Elizabethkingia meningoseptica |
| 121 | Gram-negative bacteria | Acinetobacter baumannii |
| 122 | Gram-negative bacteria | Acinetobacter calcoaceticus |
| 123 | Gram-negative bacteria | Acinetobacter ursingii |
| 124 | Gram-negative bacteria | Acinetobacter junii |
| 125 | Gram-negative bacteria | Acinetobacter nosocomialis |
| 126 | Gram-negative bacteria | Acinetobacter pittii |
| 127 | Gram-negative bacteria | Moraxella catarrhalis |
| 128 | Gram-negative bacteria | Proteus mirabilis |
| 129 | Gram-negative bacteria | Proteus vulgaris |
| 130 | Gram-negative bacteria | Proteus penneri |
| 131 | Gram-negative bacteria | Salmonella enterica |
| 132 | Gram-negative bacteria | Aggregatibacter aphrophilus |
| 133 | Gram-negative bacteria | Aggregatibacter segnis |
| 134 | Gram-negative bacteria | Bordetella pertussis |
| 135 | Gram-negative bacteria | Bartonella quintana |
| 136 | Gram-negative bacteria | Bartonella henselae |
| 137 | Gram-negative bacteria | Bartonella vinsonii |
| 138 | Gram-negative bacteria | Bartonella bacilliformis |
| 139 | Gram-negative bacteria | Bartonella elizabethae |
| 140 | Gram-negative bacteria | Providencia alcalifaciens |
| 141 | Gram-negative bacteria | Providencia stuartii |
| 142 | Gram-negative bacteria | Campylobacter fetus |
| 143 | Gram-negative bacteria | Campylobacter jejuni |
| 144 | Gram-negative bacteria | Campylobacter coli |
| 145 | Gram-negative bacteria | Citrobacter freundii |
| 146 | Gram-negative bacteria | Citrobacter braakii |
| 147 | Gram-negative bacteria | Citrobacter koseri |
| 148 | Gram-negative bacteria | Neisseria spp. |
| 149 | Gram-negative bacteria | Neisseria elongata |
| 150 | Gram-negative bacteria | Neisseria gonorrhoeae |
| 151 | Gram-negative bacteria | Neisseria mucosa |
| 152 | Gram-negative bacteria | Neisseria sicca |
| 153 | Gram-negative bacteria | Neisseria subflava |
| 154 | Gram-negative bacteria | Neisseria meningitidis |
| 155 | Gram-negative bacteria | Morganella morganii |
| 156 | Gram-negative bacteria | Vibrio vulnificus |
| 157 | Gram-negative bacteria | Pasteurella multocida |
| 158 | Gram-negative bacteria | Kingella kingae |
| 159 | Gram-negative bacteria | Serratia liquefaciens |
| 160 | Gram-negative bacteria | Serratia marcescens |
| 161 | Gram-negative bacteria | Cardiobacterium hominis |
| 162 | Gram-negative bacteria | Chryseobacterium indologenes |
| 163 | Gram-negative bacteria | Streptobacillus moniliformis |
| 164 | Gram-negative bacteria | Ochrobactrum anthropi |
| 165 | Gram-negative bacteria | Aggregatibacter actinomycetemcomitans |
| 166 | Gram-negative bacteria | Achromobacter xylosoxidans |
| 167 | Gram-negative bacteria | Edwardsiella tarda |
| 168 | Gram-negative bacteria | Bordetella trematum |
| 169 | Gram-negative bacteria | Hafnia alvei |
| 170 | Gram-negative bacteria | Eikenella corrodens |
| 171 | Gram-negative bacteria | Capnocytophaga canimorsus |
| 172 | Gram-negative bacteria | Yersinia enterocolitica |
| 173 | Gram-negative bacteria | Fusobacterium nucleatum |
| 174 | Gram-negative bacteria | Porphyromonas endodontalis |
| 175 | Gram-negative bacteria | Porphyromonas gingivalis |
| 176 | Gram-negative bacteria | Prevotella intermedia |
| 177 | Gram-negative bacteria | Fusobacterium spp. |
| 178 | Gram-negative bacteria | Fusobacterium necrophorum |
| 179 | Gram-negative bacteria | Prevotella spp. |
| 180 | Gram-negative bacteria | Veillonella spp. |
| 181 | Gram-negative bacteria | Coxiella burnetii |
| 182 | Gram-negative bacteria | Legionella pneumophila |
| 183 | Gram-negative bacteria | Prevotella melaninogenica |
| 184 | Gram-negative bacteria | Prevotella oris† |
| 185 | Gram-negative bacteria | Legionella longbeachae |
| 186 | Mycobacteria | Mycobacterium tuberculosis complex |
| 187 | Mycobacteria | Mycobacterium avium complex |
| 188 | Mycobacteria | Mycobacterium intracellulare |
| 189 | Mycobacteria | Mycobacterium abscessus |
| 190 | Mycobacteria | Mycobacterium chelonae |
| 191 | Mycobacteria | Mycobacterium genavense |
| 192 | Mycobacteria | Mycobacterium haemophilum |
| 193 | Mycobacteria | Mycobacterium kansasii |
| 194 | Mycobacteria | Mycobacterium malmoense |
| 195 | Mycobacteria | Mycobacterium marinum |
| 196 | Mycobacteria | Mycobacterium scrofulaceum |
| 197 | Mycobacteria | Mycobacterium simiae |
| 198 | Mycobacteria | Mycobacterium szulgai |
| 199 | Mycobacteria | Mycobacterium ulcerans |
| 200 | Mycobacteria | Mycobacterium flavescens |
| 201 | Mycobacteria | Mycobacterium neoaurum |
| 202 | Mycobacteria | Mycobacterium smegmatis |
| 203 | Special pathogens | Chlamydia pneumoniae |
| 204 | Special pathogens | Chlamydia psittaci |
| 205 | Special pathogens | Chlamydia trachomatis |
| 206 | Special pathogens | Rickettsia rickettsii |
| 207 | Special pathogens | Rickettsia typhi |
| 208 | Special pathogens | Rickettsia sibirica |
| 209 | Special pathogens | Mycoplasma hominis |
| 210 | Special pathogens | Mycoplasma pneumoniae |
| 211 | Special pathogens | Rickettsia prowazekii |
| 212 | Special pathogens | Anaplasma phagocytophilum |
| 213 | Special pathogens | Leptospira interrogans |
| 214 | Special pathogens | Borrelia burgdorferi |
| 215 | Special pathogens | Ureaplasma parvum |
| 216 | Special pathogens | Ureaplasma urealyticum |
| 217 | Special pathogens | Orientia tsutsugamushi |
| 218 | Special pathogens | Borrelia duttonii† |
| 219 | Special pathogens | Treponema vincentii |
| 220 | Fungi | Rhizopus spp. |
| 221 | Fungi | Rhizopus microsporus |
| 222 | Fungi | Encephalitozoon cuniculi |
| 223 | Fungi | Enterocytozoon bieneusi |
| 224 | Fungi | Rhizomucor spp. |
| 225 | Fungi | Rhizomucor pusillus |
| 226 | Fungi | Rhizomucor miehei |
| 227 | Fungi | Cunninghamella bertholletiae |
| 228 | Fungi | Aspergillus flavus |
| 229 | Fungi | Aspergillus fumigatus |
| 230 | Fungi | Aspergillus niger |
| 231 | Fungi | Aspergillus niger complex |
| 232 | Fungi | Aspergillus terreus |
| 233 | Fungi | Aspergillus versicolor |
| 234 | Fungi | Aspergillus ochraceus |
| 235 | Fungi | Aspergillus tubingensis |
| 236 | Fungi | Aspergillus lentulus |
| 237 | Fungi | Aspergillus nidulans |
| 238 | Fungi | Mucor spp. |
| 239 | Fungi | Mucor racemosus |
| 240 | Fungi | Mucor irregularis |
| 241 | Fungi | Pichia kudriavzevii (Candida krusei) |
| 242 | Fungi | Fusarium spp. |
| 243 | Fungi | Fusarium solani |
| 244 | Fungi | Fusarium graminearum |
| 245 | Fungi | Fusarium oxysporum |
| 246 | Fungi | Fusarium equiseti |
| 247 | Fungi | Candida albicans |
| 248 | Fungi | Candida auris |
| 249 | Fungi | Nakaseomyces glabratus (Candida glabrata) |
| 250 | Fungi | Candida parapsilosis |
| 251 | Fungi | Candida tropicalis |
| 252 | Fungi | Candida dubliniensis |
| 253 | Fungi | Cryptococcus neoformans |
| 254 | Fungi | Cryptococcus gattii |
| 255 | Fungi | Cryptococcus laurentii |
| 256 | Fungi | Pneumocystis jirovecii |
| 257 | Fungi | Coccidioides immitis |
| 258 | Fungi | Coccidioides posadasii |
| 259 | Fungi | Paracoccidioides brasiliensis |
| 260 | Fungi | Emergomyces orientalis |
| 261 | Fungi | (species uncertain) |
| 262 | Fungi | (species uncertain) |
| 263 | Fungi | Fonsecaea pedrosoi |
| 264 | Fungi | Exophiala dermatitidis |
| 265 | Fungi | Blastomyces dermatitidis |
| 266 | Fungi | Sporothrix schenckii |
| 267 | Fungi | Rhizopus oryzae |
| 268 | Fungi | Scedosporium apiospermum |
| 269 | Fungi | Talaromyces marneffei |
| 270 | Fungi | Histoplasma capsulatum |
| 271 | Fungi | Sporothrix globosa |
| 272 | Fungi | Lichtheimia spp. |
| 273 | Fungi | Lichtheimia corymbifera |
| 274 | Fungi | Lichtheimia ramosa |
| 275 | Fungi | Geotrichum candidum |
| 276 | Parasites | Angiostrongylus cantonensis |
| 277 | Parasites | Entamoeba histolytica |
| 278 | Parasites | Leishmania donovani |
| 279 | Parasites | Clonorchis sinensis |
| 280 | Parasites | Plasmodium malariae |
| 281 | Parasites | Plasmodium falciparum |
| 282 | Parasites | Plasmodium ovale |
| 283 | Parasites | Plasmodium vivax |
| 284 | Parasites | Taenia solium |
| 285 | Parasites | Toxoplasma gondii |
| 286 | DNA viruses | Human herpesvirus 1 (Herpes simplex virus 1, HSV-1) |
| 287 | DNA viruses | Human herpesvirus 2 (Herpes simplex virus 2, HSV-2) |
| 288 | DNA viruses | Human herpesvirus 3 (Varicella-zoster virus, VZV) |
| 289 | DNA viruses | Human herpesvirus 4 (Epstein-Barr virus, EBV) |
| 290 | DNA viruses | Human herpesvirus 5 (Cytomegalovirus, CMV) |
| 291 | DNA viruses | Human herpesvirus 6 (HHV-6) |
| 292 | DNA viruses | Human polyomavirus 1 (BK virus) |
| 293 | DNA viruses | Human polyomavirus 2 (JC virus) |
| 294 | DNA viruses | Human bocavirus |
| 295 | DNA viruses | Human bocavirus 1 |
| 296 | DNA viruses | Human bocavirus 2 |
| 297 | DNA viruses | Primate erythroparvovirus 1 (Parvovirus B19) |
| 298 | DNA viruses | Human mastadenovirus A |
| 299 | DNA viruses | Human mastadenovirus B |
| 300 | DNA viruses | Human mastadenovirus C |
| 301 | DNA viruses | Human mastadenovirus D |
| 302 | DNA viruses | Human mastadenovirus E |
| 303 | DNA viruses | Human mastadenovirus F |
| 304 | DNA viruses | Human mastadenovirus G |
| 305 | DNA viruses | Human adenovirus type 3 |
| 306 | DNA viruses | Human adenovirus type 7 |
| 307 | DNA viruses | Human adenovirus type 11 |
| 308 | DNA viruses | Human adenovirus type 14 |
| 309 | DNA viruses | Human adenovirus type 21 |
| 310 | DNA viruses | Human adenovirus type 55 |
| 311 | DNA viruses | Human adenovirus type 1 |
| 312 | DNA viruses | Human adenovirus type 2 |
| 313 | DNA viruses | Human adenovirus type 5 |
| 314 | DNA viruses | Human adenovirus type 6 |
| 315 | DNA viruses | Human adenovirus type 4 |
| 316 | DNA viruses | Mpox virus (Monkeypox virus) |
| 317 | RNA viruses | Rhinovirus A |
| 318 | RNA viruses | Rhinovirus B |
| 319 | RNA viruses | Rhinovirus C |
| 320 | RNA viruses | Human metapneumovirus (hMPV) |
| 321 | RNA viruses | Human coronavirus 229E |
| 322 | RNA viruses | Human coronavirus HKU1 |
| 323 | RNA viruses | Human coronavirus NL63 |
| 324 | RNA viruses | Human coronavirus OC43 |
| 325 | RNA viruses | Human respiratory syncytial virus A (RSV-A) |
| 326 | RNA viruses | Human respiratory syncytial virus B (RSV-B) |
| 327 | RNA viruses | Human parainfluenza virus type 1 |
| 328 | RNA viruses | Human parainfluenza virus type 3 |
| 329 | RNA viruses | Influenza A virus |
| 330 | RNA viruses | Influenza A virus, H1N1 subtype |
| 331 | RNA viruses | Influenza A virus, H3N2 subtype |
| 332 | RNA viruses | Influenza A virus, H5N1 subtype |
| 333 | RNA viruses | Influenza A virus, H7N9 subtype |
| 334 | RNA viruses | Influenza B virus |
| 335 | RNA viruses | Influenza B virus, Victoria lineage |
| 336 | RNA viruses | Influenza B virus, Yamagata lineage |
| 337 | RNA viruses | Influenza C virus |
| 338 | RNA viruses | Human parainfluenza virus type 2 |
| 339 | RNA viruses | Human parainfluenza virus type 4 |
| 340 | RNA viruses | Mumps virus |
| 341 | RNA viruses | SARS-CoV-2 (novel coronavirus) |
| 342 | RNA viruses | SARS-CoV |
| 343 | RNA viruses | MERS-CoV |
| 344 | RNA viruses | Japanese encephalitis virus |
| 345 | RNA viruses | Human T-lymphotropic virus (HTLV) |
| 346 | RNA viruses | Human T-lymphotropic virus type 1 (HTLV-1) |
| 347 | RNA viruses | Human T-lymphotropic virus type 2 (HTLV-2) |
| 348 | RNA viruses | Norovirus |
| 349 | RNA viruses | Dengue virus |
| 350 | RNA viruses | Dengue virus type 1 |
| 351 | RNA viruses | Dengue virus type 2 |
| 352 | RNA viruses | Dengue virus type 3 |
| 353 | RNA viruses | Dengue virus type 4 |
| 354 | RNA viruses | Hantaan virus |
| 355 | RNA viruses | Seoul virus |
| 356 | RNA viruses | Severe fever with thrombocytopenia syndrome virus (SFTSV) |
| 357 | RNA viruses | Enterovirus A |
| 358 | RNA viruses | Enterovirus B |
| 359 | RNA viruses | Enterovirus C |
| 360 | RNA viruses | Enterovirus D |
| 361 | RNA viruses | Enterovirus D68 |
| 362 | RNA viruses | Echovirus (Species A) |
| 363 | RNA viruses | Echovirus (Species B) |
| 364 | RNA viruses | Echovirus (Species C) |
| 365 | RNA viruses | Echovirus (Species D) |
| 366 | RNA viruses | Coxsackievirus A6 |
| 367 | RNA viruses | Coxsackievirus A10 |
| 368 | RNA viruses | Coxsackievirus A16 |
| 369 | RNA viruses | Coxsackievirus B3 |
| 370 | RNA viruses | Coxsackievirus B5 |
| 371 | RNA viruses | Rotavirus A |
| 372 | RNA viruses | Rubella virus |
| 373 | RNA viruses | Measles virus |
| 374 | RNA viruses | Chikungunya virus |
| 375 | RNA viruses | Tick-borne encephalitis virus |
| 376 | RNA viruses | Lymphocytic choriomeningitis virus |
| 377 | RNA viruses | Rabies virus |
| 378 | RNA viruses | Sapovirus |
| 379 | RNA viruses | Yellow fever virus |
| 380 | RNA viruses | Zika virus |
| 381 | RNA viruses | Mamastrovirus 1 (Human astrovirus 1) |
| 382 | RNA viruses | Enterovirus A71 |
| 383 | RNA viruses | Echovirus 11 |

Section B. Antimicrobial Resistance Genes (n = 55)

| **No.** | **Drug Class** | **Resistance Gene** | **Related Drug(s)** |
| --- | --- | --- | --- |
| 1 | Aminoglycosides | AAC(6')-Ie-APH(2")-Ia bifunctional protein | Aminoglycosides (neomycin, amikacin, gentamicin, etc.) |
| 2 | Aminoglycosides | armA | Aminoglycosides (amikacin, gentamicin, etc.) |
| 3 | Aminoglycosides | npmA | Aminoglycosides (neomycin, gentamicin, etc.) |
| 4 | Aminoglycosides | rmtA | Aminoglycosides (amikacin, gentamicin, etc.) |
| 5 | Aminoglycosides | rmtB | Aminoglycosides (amikacin, gentamicin, etc.) |
| 6 | Aminoglycosides | rmtC | Aminoglycosides (amikacin, gentamicin, etc.) |
| 7 | Aminoglycosides | rmtD | Aminoglycosides (amikacin, gentamicin, etc.) |
| 8 | Aminoglycosides | rmtE | Aminoglycosides (amikacin, gentamicin, etc.) |
| 9 | β-lactams (mecA/mecC-mediated) | mecA | β-lactams (excluding 5th-generation cephalosporins) |
| 10 | β-lactams (mecA/mecC-mediated) | mecC | β-lactams (excluding 5th-generation cephalosporins) |
| 11 | Macrolide-Lincosamide-Streptogramin B (MLSB) | ermA | MLSB agents (erythromycin, clindamycin, etc.) |
| 12 | Macrolide-Lincosamide-Streptogramin B (MLSB) | ermB | MLSB agents (erythromycin, clindamycin, etc.) |
| 13 | Sulfonamides | sul1 | Sulfonamides (sulfamethoxazole, etc.) |
| 14 | Sulfonamides | sul2 | Sulfonamides (sulfamethoxazole, etc.) |
| 15 | Fluoroquinolones | qnrA | Fluoroquinolones (ciprofloxacin, levofloxacin, etc.) |
| 16 | Fluoroquinolones | qnrB | Fluoroquinolones (ciprofloxacin, levofloxacin, etc.) |
| 17 | Glycopeptides | vanA | Glycopeptides (vancomycin, teicoplanin) |
| 18 | Glycopeptides | vanB | Glycopeptides (vancomycin) |
| 19 | Tetracyclines | tet(M) | Tetracyclines (doxycycline, minocycline, etc.) |
| 20 | Tetracyclines | tet(O) | Tetracyclines (doxycycline, minocycline, etc.) |
| 21 | Tetracyclines | tet(X3) | Tigecycline |
| 22 | Polymyxins | mcr-1 | Polymyxins (colistin) |
| 23 | Penicillins | bro-1 | Penicillins (ampicillin, amoxicillin, etc.) |
| 24 | Extended-spectrum β-lactamases (ESBL) | blaCTX-M | Penicillins, cephalosporins, monobactams |
| 25 | Extended-spectrum β-lactamases (ESBL) | blaPER | Penicillins, cephalosporins, monobactams |
| 26 | Extended-spectrum β-lactamases (ESBL) | blaSHV | Penicillins, cephalosporins, monobactams |
| 27 | Extended-spectrum β-lactamases (ESBL) | blaTEM | Penicillins, cephalosporins, monobactams |
| 28 | Extended-spectrum β-lactamases (ESBL) | blaGES | Carbapenems, penicillins, cephalosporins, monobactams |
| 29 | Carbapenemases, Class A | blaKPC | Carbapenems, penicillins, cephalosporins, monobactams |
| 30 | Carbapenemases, Class A | blaSME | Carbapenems, penicillins, cephalosporins, monobactams |
| 31 | Carbapenemases, Class A | blaIMI | Carbapenems, penicillins, cephalosporins, monobactams |
| 32 | Carbapenemases, Class B (metallo-β-lactamases) | blaGIM | Carbapenems, cephalosporins, penicillins, cephamycins |
| 33 | Carbapenemases, Class B (metallo-β-lactamases) | blaIMP | Carbapenems, cephalosporins, penicillins, cephamycins |
| 34 | Carbapenemases, Class B (metallo-β-lactamases) | blaNDM | Carbapenems, cephalosporins, penicillins, cephamycins |
| 35 | Carbapenemases, Class B (metallo-β-lactamases) | blaSIM | Carbapenems, cephalosporins, penicillins, cephamycins |
| 36 | Carbapenemases, Class B (metallo-β-lactamases) | blaSPM | Carbapenems, cephalosporins, penicillins, cephamycins |
| 37 | Carbapenemases, Class B (metallo-β-lactamases) | blaVIM | Carbapenems, cephalosporins, penicillins, cephamycins |
| 38 | Carbapenemases, Class D | blaOXA-23 | Carbapenems, penicillins, cephalosporins |
| 39 | Carbapenemases, Class D | blaOXA-48 | Carbapenems, penicillins, cephalosporins |
| 40 | AmpC β-lactamases | blaACC | Penicillins, cephamycins, cephalosporins, monobactams |
| 41 | AmpC β-lactamases | blaACT | Penicillins, cephamycins, cephalosporins, monobactams |
| 42 | AmpC β-lactamases | blaADC | Penicillins, cephamycins, cephalosporins, monobactams |
| 43 | AmpC β-lactamases | blaCMY | Penicillins, cephamycins, cephalosporins, monobactams |
| 44 | AmpC β-lactamases | blaDHA | Penicillins, cephamycins, cephalosporins, monobactams |
| 45 | AmpC β-lactamases | blaFOX | Penicillins, cephamycins, cephalosporins, monobactams |
| 46 | AmpC β-lactamases | blaMOX | Penicillins, cephamycins, cephalosporins, monobactams |
| 47 | AmpC β-lactamases | blaMIR | Penicillins, cephamycins, cephalosporins, monobactams |
| 48 | Macrolide resistance (23S rRNA mutations) | 23S rRNA (A2063G) | 14-membered-ring macrolides (erythromycin, etc.) |
| 49 | Macrolide resistance (23S rRNA mutations) | 23S rRNA (A2064G) | 14- and 16-membered-ring macrolides (erythromycin, josamycin, etc.) |
| 50 | Macrolide resistance (23S rRNA mutations) | 23S rRNA (A2067G) | 16-membered-ring macrolides (josamycin, etc.) |
| 51 | Macrolide resistance (23S rRNA mutations) | 23S rRNA (C2617G) | 14- and 15-membered-ring macrolides (erythromycin, azithromycin) |
| 52 | Macrolide resistance (23S rRNA mutations) | 23S rRNA (A2047G) | 14- and 15-membered-ring macrolides (erythromycin, azithromycin) |
| 53 | Macrolide resistance (23S rRNA mutations) | 23S rRNA (A2330T) | 14- and 15-membered-ring macrolides (erythromycin, azithromycin) |
| 54 | Azole resistance | ERG11 | Azoles (fluconazole, voriconazole, etc.) |
| 55 | Azole resistance | cyp51A | Azoles (fluconazole, voriconazole, etc.) |

Section C. Virulence Factors (n = 9)

| **No.** | **Species** | **Virulence Factor** |
| --- | --- | --- |
| 1 | Klebsiella pneumoniae | RmpA |
| 2 | Klebsiella pneumoniae | Aerobactin |
| 3 | Klebsiella pneumoniae | Sal |
| 4 | Staphylococcus aureus | Coagulase |
| 5 | Staphylococcus aureus | PVL (Panton-Valentine leukocidin) |
| 6 | Acinetobacter baumannii | OmpA |
| 7 | Pseudomonas aeruginosa | ExoA |
| 8 | Pseudomonas aeruginosa | TTSS secreted effectors |
| 9 | Streptococcus pneumoniae | Pneumolysin |

### Table S4. Baseline characteristics and diagnostic performance by enrollment center

| **Characteristic** | **Center 1 (n=57)** | **Center 2 (n=36)** | **P value** |
| --- | --- | --- | --- |
| **Demographics and disease severity** | | | |
| Age, years | 70.0 [58.0–76.0] | 70.5 [61.5–74.0] | 0.819 |
| Female sex, n (%) | 11 (19.3) | 14 (38.9) | 0.054 |
| APACHE II score | 14.0 [11.0–19.0] | 14.5 [10.0–20.5] | 0.956 |
| SOFA score | 6.0 [4.0–10.0] | 5.0 [2.8–9.2] | 0.412 |
| **Comorbidities, n (%)** | | | |
| Heart failure | 12 (21.1) | 12 (33.3) | 0.227 |
| Diabetes mellitus | 20 (35.1) | 13 (36.1) | 1.000 |
| Chronic kidney disease | 12 (21.4) | 7 (19.4) | 1.000 |
| Malignancy | 24 (42.1) | 11 (30.6) | 0.282 |
| Autoimmune disease | 2 (3.5) | 2 (5.6) | 0.639 |
| Prior antibiotic exposure, n (%) | 45 (78.9) | 33 (91.7) | 0.149 |
| **Immune status** | | | |
| Total lymphocytes, ×10⁹/L^a^ | 0.6 [0.5–0.9] | 0.6 [0.4–0.9] | 0.837 |
| T cells, cells/mm³^a^ | 319.0 [215.0–484.0] | 336.0 [206.2–491.2] | 0.720 |
| CD4+ T cells, cells/mm³^a^ | 178.0 [108.0–274.0] | 172.0 [113.0–274.2] | 0.881 |
| CD8+ T cells, cells/mm³^a^ | 109.0 [66.0–178.0] | 131.0 [66.8–202.2] | 0.492 |
| B cells, cells/mm³^a^ | 83.0 [28.0–138.0] | 94.0 [33.0–158.8] | 0.699 |
| NK cells, cells/mm³^a^ | 45.0 [34.0–87.0] | 43.5 [16.5–66.0] | 0.468 |
| **tNGS performance** | | | |
| Overall diagnostic concordance, n/N (%)^b^ | 38/46 (82.6) | 19/27 (70.4) | 0.252 |
| Turnaround time, days | 1.0 ᶜ | — | — |

Abbreviations: IQR, interquartile range; APACHE II, Acute Physiology and Chronic Health Evaluation II; SOFA, Sequential Organ Failure Assessment; CD4+, CD4-positive T-lymphocyte; tNGS, targeted next-generation sequencing. Turnaround time was uniformly 1 day for 93 patients, precluding between‑center comparison; data from both centers are reported jointly.

**Table S5. Positivity rates of tNGS versus CMT by specimen type, with Wilson 95% confidence intervals and paired McNemar's test**

| **Specimen type** | **tNGS positive, n/N (%)** | **tNGS 95% CI** | **CMT positive, n/N (%)** | **CMT 95% CI** | **McNemar's P** |
| --- | --- | --- | --- | --- | --- |
| Blood/Plasma | 17/27 (63.0%) | 44.2–78.5% | 16/26 (61.5%) | 42.5–77.6% | >0.99 |
| Sputum | 25/25 (100%) | 86.7–100% | 20/25 (80.0%) | 60.9–91.1% | 0.074 |
| BALF | 24/24 (100%) | 86.2–100% | 21/24 (87.5%) | 69.0–95.7% | 0.248 |
| CSFᵃ | 0/2 (0%) | 0–65.8% | 1/2 (50.0%) | 9.5–90.5% | >0.99 |
| Other effusion/drainage | 14/15 (93.3%) | 70.2–98.8% | 11/14 (78.6%) | 52.4–92.4% | 0.248 |

*tNGS, targeted next-generation sequencing; CMT, conventional microbiological testing; CI, confidence interval. Positivity rates and 95% CIs were calculated using the Wilson score method. Within-patient concordance between tNGS and CMT positivity was assessed using paired McNemar's test for each specimen type. No significant differences were observed for any specimen type (all P ≥ 0.07).*

*ᵃ Given the very small CSF sample size (n = 2), this result should be interpreted with caution.*
